# *GSTP1* and *GSTM3* Variant Alleles Affect Susceptibility and Severity of COVID-19

**DOI:** 10.3389/fmolb.2021.747493

**Published:** 2021-12-20

**Authors:** Vesna Coric, Ivana Milosevic, Tatjana Djukic, Zoran Bukumiric, Ana Savic-Radojevic, Marija Matic, Djurdja Jerotic, Nevena Todorovic, Milika Asanin, Marko Ercegovac, Jovan Ranin, Goran Stevanovic, Marija Pljesa-Ercegovac, Tatjana Simic

**Affiliations:** ^1^ Faculty of Medicine, University of Belgrade, Belgrade, Serbia; ^2^ Institute of Medical and Clinical Biochemistry, Belgrade, Serbia; ^3^ Clinic of Infectious and Tropical Diseases, Clinical Centre of Serbia, Belgrade, Serbia; ^4^ Institute of Medical Statistics and Informatics, Belgrade, Serbia; ^5^ Clinic of Neurology, Clinical Centre of Serbia, Belgrade, Serbia; ^6^ Clinic of Cardiology, Clinical Centre of Serbia, Belgrade, Serbia; ^7^ Serbian Academy of Sciences and Arts, Belgrade, Serbia

**Keywords:** oxidative stress, COVID-19, glutathione transferases, GSTP1, GSTM3, polymorphisms

## Abstract

Based on the premise that oxidative stress plays an important role in severe acute respiratory syndrome coronavirus (SARS-CoV-2) infection, we speculated that variations in the antioxidant activities of different members of the glutathione S-transferase family of enzymes might modulate individual susceptibility towards development of clinical manifestations in COVID-19. The distribution of polymorphisms in cytosolic glutathione S-transferases *GSTA1*, *GSTM1*, *GSTM3*, *GSTP1* (*rs1695* and *rs1138272*), and *GSTT1* were assessed in 207 COVID-19 patients and 252 matched healthy individuals, emphasizing their individual and cumulative effect in disease development and severity. *GST* polymorphisms were determined by appropriate PCR methods. Among six *GST* polymorphisms analyzed in this study, *GSTP1* rs1695 and *GSTM3* were found to be associated with COVID-19. Indeed, the data obtained showed that individuals carrying variant *GSTP1*-*Val* allele exhibit lower odds of COVID-19 development (*p* = 0.002), contrary to carriers of variant *GSTM3-CC* genotype which have higher odds for COVID-19 (*p* = 0.024). Moreover, combined *GSTP1* (*rs1138272* and *rs1695*) and *GSTM3* genotype exhibited cumulative risk regarding both COVID-19 occurrence and COVID-19 severity (*p* = 0.001 and *p* = 0.025, respectively). Further studies are needed to clarify the exact roles of specific glutathione S-transferases once the SARS-CoV-2 infection is initiated in the host cell.

## Introduction

It has been suggested that oxidative stress plays an important role in severe acute respiratory syndrome coronavirus (SARS-CoV-2) infection. Namely, oxidative stress is supposed to mediate various processes in COVID-19, including binding to viral receptor and replication, enhanced cytokine production, inflammation, and cell signaling (Fernandes et al., 2020). Reactive oxygen species (ROS)-induced activation of transcription factors and pro-inflammatory genes triggers immune cells to secrete various cytokines and chemokines, leading to additional ROS generation by immune cells (Chatterjee, 2016). Moreover, redox-sensitive p38 mitogen-activated protein kinases (MAPK) pathway is shown to be induced in COVID-19, shedding even more light on the importance of disturbed redox homeostasis in SARS-CoV-2 infection (Cheng et al., 2020). It seems that both overproduction of ROS and failure of antioxidant mechanisms affect viral replication and accompanying clinical manifestations of virus-associated disease. Furthermore, tissue damage consequential to ROS overproduction reflects the severity of viral infection (Naumenko et al., 2018). Still, the data on the antioxidant defense system, as an important determinant of redox balance, are scarce. In the view of significant variations in response to SARS-CoV-2 infection, co-morbidities, and suspected role of oxidative stress, it is biologically plausible that inter-individual differences in susceptibility, as well as severity of clinical manifestations in COVID-19 patients, might be affected by their antioxidant genetic profile.

Members of the glutathione S-transferase (GST) superfamily, which are known for detoxifying xenobiotics, also exhibit diverse antioxidant and anti-inflammatory roles (Tew and Townsend, 2012; Menon et al., 2017; Pljesa-Ercegovac et al., 2018). Additionally, the involvement of several GSTs in the regulation of signaling pathways, by means of interactions with members of the MAPK signaling pathway (JNK-c-Jun N-terminal kinase, ASK-apoptosis signal-regulating kinase, Akt-protein kinase B) and certain receptors, is well established (Pljesa-Ercegovac et al., 2018). The most important cytosolic GST comprises alpha (GSTA), mi (GSTM), pi (GSTP), and theta (GSTT) classes. Deletion polymorphism in *GSTM1* and *GSTT1* genes leads to complete lack of enzymatic activity in homozygous carriers of the *null* genotype. Additionally, *GSTM3* and *GSTA1* polymorphisms influence gene expression, while two linked *GSTP1* single-nucleotide polymorphisms modify substrate specificity.

So far, it has been revealed that *GSTT1*- and *GSTM1*-*null* genotypes have differential behavior versus COVID-19 mortality. Namely, individuals with lower frequency of the *GSTT1-null* genotype exhibit higher COVID-19 mortality (Saadat, 2020a; Abbas et al., 2021) which is important considering the fact that *GST* genotypes distribution is ethnicity-dependent (Polimanti et al., 2013). Similarly, morbidity and mortality of COVID-19 also correlate with *GSTP1*-*Ile105Val* polymorphism in a way that countries with more frequent *Val105* allele have higher prevalence and mortality of COVID-19 (Saadat, 2020b). The potential effect of *GSTP1* in COVID-19 should also be analyzed in line with its anti-inflammatory role (Zhou et al., 2018). Surprisingly, the role of established antioxidant GST member, GSTA1, has not been evaluated as yet.

Since genetic polymorphisms in the antioxidant defense system are recognized as determinants of risk and prognosis of the major COVID-19 comorbidities, their role in susceptibility to development of COVID-19 clinical manifestations should be clarified. In this line, we aimed to assess the distribution of polymorphisms in genes encoding glutathione transferases alpha (*GSTA1*), mu (*GSTM1*, *GSTM3*), pi (*GSTP1* rs1695 and *GSTP1* rs1138272), and theta (*GSTT1*) in COVID-19 patients and matched healthy individuals, emphasizing their individual and cumulative effect in disease development.

## Materials and Methods

### Study Group

The study group comprised 207 COVID-19 patients (120 men and 87 women, with an average age of 52.9 ± 13.9 years) treated at the Institute of Infectious and Tropical Diseases, Clinical Centre of Serbia, between July 2020 and February 2021. Inclusion criteria for participation in the study were as follows: positive SARS-CoV-2 reverse transcription (RT)-PCR test performed from nasopharyngeal and oropharyngeal swabs according to World Health Organization guidelines and using available RT-PCR protocols, age (≥18 years old), and their willingness to provide written informed consent. Age- and gender-matched control group included 252 individuals (123 men, 129 women; average age 51.5 ± 13.0 years) with confirmed lack of SARS-CoV-2 antibodies (IgM and IgG). The controls were randomly chosen among subjects exposed to the same infection risks as the patient group in order to obtain the groups of homogeneous origin. All participants were Caucasians by ethnicity.

The principles of International Conference on Harmonisation (ICH) Good Clinical Practice, the “Declaration of Helsinki,” and national and international ethical guidelines were followed during this study with approval obtained from the Ethics Committee of Clinical Centre of Serbia (566/01 from July 13, 2020 and 608/01 from August 7, 2020). Informed written consent was procured from all recruited subjects.

### DNA Isolation and Glutathione Transferases Genotyping

A total DNA was purified from EDTA-anticoagulated peripheral blood obtained from the study participants using PureLink™ Genomic DNA Mini Kit (*ThermoFisher Scientific*, United States).

*GSTP1 rs1695*, *GSTP1 rs1138272*, and *GSTM3 rs1332018* polymorphisms were determined by real-time PCR on Mastercycler ep realplex (*Eppendorf*, Germany), using TaqMan Drug Metabolism Genotyping assays (*Life Technologies*, *Applied Biosystems*, United States). Assays' IDs were as follows: C_3237198_20, C_1049615_20, and C_3184522_30, respectively.

*GSTM1* and *GSTT1* deletion polymorphisms were determined by multiplex PCR, using *CYP1A1* gene as an internal control. *GSTA1* rs3957357 polymorphism was determined by PCR-restriction fragment length polymorphism (PCR-RFLP). Enzymatic digestion of amplified sequence was performed overnight at 37°C using *EarI* restriction enzyme (*Thermo Fisher Scientific*, United States). Primers sequences of *GSTM1*, *GSTT1*, *CYP1A1*, and *GSTA1* genes and PCR protocols details are given in Table 1. PCR products were separated on 2% agarose gel stained with SYBR^®^ Safe DNA Gel Stain (*Invitrogen*, United States) and visualized on Chemidoc (*Biorad*, United States).

**TABLE 1:** Primers sequences of *GSTM1*, *GSTT1*, *CYP1A1*, and *GSTA1* genes, and PCR protocol details.

Gene	Primer sequence	PCR protocol	PCR products
*GSTA1 rs3957357*	F: 5′- GCATCAGCT TGCCCTTCA-3′, R: 5′-AAA​CGC​TGT​CAC​CGT​CCT-3′	RFLP PCR	*GSTA1 C/C:* 481 bp, *GSTA1 C/T*, 481 bp, 385 bp and 96 bp, *GSTA1 T/T*, 385 bp, and 96 bp
		Denature: 95°C for 1 min	
		Followed by 94°C for 1 min; annealing: 62°C for 1 min	
		Extension: 72°C for 1 min	
		Final extension: 72°C for 7 min	
		31 cycles	
*GSTM1* deletion	F: 5′-GAA​CTC​CCT​GAA​AAG​CTA​AAG​C-3′ R: 5′-GTT​GGG​CTC​AAA​TAT​ACG​GTG​G-3′	Multiplex PCR, denature: 94°C for 3 min, followed by 94°C for 30 s; annealing: 59°C for 30 s; extension: 72°C for 45 s; final extension: 72°C for 4 mi, 35 cycles	*GSTM1-active*: 215 bp
*GSTT1* deletion	F: 5′-TTC​CTT​ACT​GGT​CCT​CAC​ATC​TC-3′ R: 5′-TCA​CGG​GAT​CAT​GGC​CAG​CA-3′		*GSTT1-active*: 480 bp
*CYP1A1* positive control	F: 5′-GAACTGCCACTT CAGCTGTCT-3′ R: 5′-CAG​CTG​CAT​TTG​GAA​GTG​CTC-3′		312 bp

### Statistical Analysis

Statistical data analysis was performed using IBM SPSS Statistics 22 (*SPSS* Inc., Chicago, IL, United States). Results were presented as frequency, percent and mean ± SD. Data were analyzed using univariate and multivariate logistic regression for calculating odds ratio (OR) and 95% confidence interval (95%CI) in order to determine the potential association between *GST* genotypes and odds for the development of COVID-19. Age, gender, presence of diabetes mellitus type 2, hypertension, obesity, and smoking habits were considered as confounding factors in analysis. All *p*-values less than 0.05 were considered significant.

## Results

A total of 207 COVID-19 patients and 252 age- and gender-matched controls were included in the study. Baseline characteristics of patients and controls are summarized in Table 2. As presented, most frequent COVID-19 comorbidities, such as diabetes, obesity, and hypertension, were evaluated. No significant difference among groups was found regarding these most frequent comorbidities. However, almost 50% of controls were smokers in comparison with 16% of smokers in the COVID-19 group (*p* < 0.001). Out of 43% of patients who needed hospitalization, none required mechanical ventilation.

**TABLE 2:** Baseline characteristic of 207 COVID-19 patients and 252 age- and gender-matched controls.

	COVID-19 patients	Controls	OR (95%CI)	*p*-value
Age (years)^a^	52.9 ± 13.9	51.5 ± 13.0	1.03 (0.99–1.05)	0.071
Gender, n (%)				
Male	120 (58)	129 (53)	1.00^b^	
Female	87 (42)	123 (47)	0.70 (0.41–1.20)	0.196
Hypertension, n (%)				
No	122 (59)	179 (71)	1.00^b^	
Yes	85 (41)	73 (29)	1.19 (0.63–2.24)	0.585
Obesity, n (%)				
BMI < 30	142 (69)	207 (82)	1.00^b^	
BMI > 30	65 (31)	45 (18)	1.74 (0.95–3.18)	0.072
BMI (kg/m^2^)^a^	28.3 ± 4.9	26.3 ± 4.3	1.05 (0.99–1.15)	0.117
Smoking, n (%)				
Never	102 (49)	93 (37)	1.00^b^	
Former	73 (35)	35 (14)	1.69 (0.88–3.22)	0.114
Ever	32 (16)	124 (49)	0.29 (0.15–0.55)	<0.001
Diabetes				
No	188 (91)	234 (93)	1.00^b^	
Yes	19 (9)	18 (7)	1.58 (0.58–4.28)	0.367

a Mean ± SD.

b Reference group; CI, confidence interval.

The distribution of specific genotypes among COVID-19 patients and controls is presented in Table 3. Among six investigated GST polymorphisms, significant association between GST genotype and susceptibility for development of COVID-19 clinical manifestations was found for both GSTP1 (rs 1695 and rs1138272) and GSTM3 (rs1332018) polymorphisms. Namely, carriers of heterozygous GSTP1 *IleVal* rs1695 genotype are less prone to develop COVID-19 (OR = 0.66, 95%CI = 0.44–0.98, *p* = 0.042). Similarly, individuals with at least one GSTP1* *Val* allele rs1138272 had significantly lower odds of COVID-19 development (*p* < 0.05) compared to the carriers of wild-type GSTP1 *AlaAla* genotype. As for GSTM3 polymorphism, carriers of GSTM3 *AC* genotype had significantly lower odds of developing COVID-19 compared to individuals with GSTM3 *AA* genotype (OR = 0.60, 95%CI = 0.38–0.96, *p* = 0.033), while homozygous carriers of GSTM3*C allele had 1.7-fold increased COVID-19 odds but with borderline significance (OR = 1.71, 95%CI = 0.99–2.95, *p* = 0.053).

**TABLE 3:** The distribution of specific *GST* genotypes among COVID-19 patients and controls.

*GST* genotype	COVID-19 patients n, %	Controls n, %	OR (95%CI)^c^	*p*-value
*GSTM1*				
*Active* ^a^	93 (45)	114 (49)	1.00^d^	
*Null* ^b^	114 (55)	117 (51)	1.19 (0.82–1.74)	0.355
*GSTT1*				
*active* ^a^	163 (79)	187 (81)	1.00^d^	
*null* ^b^	44 (21)	44 (19)	1.15 (0.72–1.83)	0.565
*GSTA1* (*rs3957357*)				
*CC* (*active*)	69 (33)	76 (33)	1.00^d^	
*CT*	98 (47)	110 (48)	0.98 (0.64–1.50)	0.931
*TT*	40 (19)	44 (19)	1.00 (0.59–1.71)	0.995
*GSTP1* (*rs1695*)				
*IleIle* (*wild-type*)	89 (45)	79 (34)	1.00^d^	
*IleVal*	90 (46)	122 (53)	0.66 (0.44–0.98)	0.042
*ValVal*	17 (9)	28 (12)	0.54 (0.28–1.06)	0.072
*GSTP1* (*rs1138272*)				
*AlaAla* (*wild-type*)	157 (76)	141 (54)	1.00^d^	
*AlaVal*	48 (23)	68 (31)	0.63 (0.41–0.99)	0.039
*ValVal*	1 (1)	11 (5)	0.08 (0.10–0.64)	0.017
*GSTM3* (*rs1332018*)				
*AA*	72 (38)	68 (36)	1.00^d^	
*AC*	58 (31)	91 (48)	0.60 (0.38–0.96)	0.033
*CC*	58 (31)	32 (17)	1.71 (0.99–2.95)	0.053

a *Active*, if at least one active allele present.

b *Null* if no active alleles present.

c OR, crude odds ratio; CI, confidence interval.

d Reference group.

When GST genotypes distribution in COVID-19 patients and controls was assessed including adjustment for age, gender, smoking habit, and comorbidities, comprising hypertension, obesity, and diabetes, significant association remained for GSTP1 (rs1695) and GSTM3 (rs1332018) polymorphisms (Table 4). Namely, we observed that individuals with GSTP1 *IleVal* rs 1695 genotype were almost 3-fold less prone for COVID-19 development (OR = 0.33, 95%CI = 0.17–10.67, *p* = 0.002) in comparison to the carriers of wild-type GSTP1 *IleIle* genotype. On the contrary, carriers of *GSTM3CC* genotype had 2.5-fold higher odds compared to the carriers of *GSTM3AA* genotype (OR = 2.51, 95%CI = 1.12–5.61, *p* = 0.024).

**TABLE 4:** The distribution of *GST* genotypes among COVID-19 patients and controls adjusted for age, gender, comorbidities, and smoking.

*GST* genotype	COVID-19 patients n, %	Controls n, %	OR (95%CI)^c^	*p*-value
*GSTM1*				
*active* ^a^	93 (45)	114 (49)	1.00^d^	
*null* ^b^	114 (55)	117 (51)	1.09 (0.49–1.62)	0.692
*GSTT1*				
*active* ^a^	163 (79)	187 (81)	1.00^d^	
*null* ^b^	44 (21)	44 (19)	1.32 (0.63–2.75)	0.462
*GSTA1* (*rs3957357*)				
*CC* (*active*)	69 (33)	76 (33)	1.00^d^	
*CT*	98 (47)	110 (48)	0.93 (0.47–1.84)	0.829
*TT*	40 (19)	44 (19)	1.43 (0.64–3.23)	0.387
*GSTP1* (*rs1695*)				
*IleIle* (*wild-type*)	89 (45)	79 (34)	1.00^d^	
*IleVal*	90 (46)	122 (53)	0.34 (0.17–0.67)	0.002
*ValVal*	17 (9)	28 (12)	0.50 (0.14–1.81)	0.293
*GSTP1* (*rs1138272*)				
*AlaAla* (*wild-type*)	157 (76)	141 (54)	1.00^d^	
*AlaVal*	48 (23)	68 (31)	0.89 (0.44–1.79)	0.744
*ValVal*	1 (1)	11 (5)	0.21 (0.02–2.44)	0.211
*GSTM3* (*rs1332018*)				
*AA*	72 (38)	68 (36)	1.00^d^	
*AC*	58 (31)	91 (48)	0.73 (0.38–0.1.44)	0.367
*CC*	58 (31)	32 (17)	2.52 (1.13–5.61)	0.024

a *Active*, if at least one active allele present.

b *Null* if no active alleles present.

c OR, odds ratio adjusted for gender, age, hypertension, diabetes mellitus, smoking and obesity; CI, confidence interval.

d Reference group.

Finally, the cumulative effect of COVID-19 risk-associated GST genotypes was analyzed in a binary logistic model adjusted for age, gender, comorbidities, and smoking habit. As presented in Table 5, carriers of one, two, or three risk-associated GST genotypes in comparison to the referent category, comprising none of the risk-associated genotypes, exhibited statistically significant increase in odds towards COVID-19. Namely, a trend in OR was observed in COVID-19 patients with one risk-associated genotype, OR = 2.76, *p* = 0.012, with two risk-associated genotypes, OR = 3.38, *p* = 0.002, while the highest OR was found in patients with three risk-associated genotypes, OR = 11.86, *p* = 0.001. Furthermore, the overall analysis of the number of risk-related *GST* genotypes was found to be statistically significant (OR = 1.88, 95%CI = 1.35–2.61, *p* < 0.001).

**TABLE 5:** Cumulative effect of COVID-19 risk-associated *GST* genotypes.

Number of risk-associated *GST* genotypes	COVID-19 patients n (%)	Controls n (%)	OR (95%CI)^a^	*p*-value
0	23 (11.2)	67 (28.8)	1^b^	
1	83 (40.3)	83 (35.8)	2.76 (0.25–6.07)	0.012
2	79 (38.3)	77 (33.2)	3.38 (1.56–7.34)	0.002
3	21 (10.2)	5 (2.2)	11.86 (2.84–49.40)	0.001

0: Reference genotype combination carrying lowest odds (*GSTP1-ValVal/GSTP1-ValVal*, *GSTM3-AA*); 1, 2, 3: The number of risk-associated alleles: either one, two or three risk-associated GST, genotypes (comprising GSTP1*Ile or GSTP1*Ala or GSTM3*C).

a OR, odds ratio adjusted for age, gender, hypertension, diabetes mellitus, smoking and obesity.

b Reference group; CI, confidence interval.

Besides, the cumulative effect of *GST* genotypes was also analyzed in terms of the disease severity. Namely, grouping patients with mild symptoms vs. severe symptoms enabled us to evaluate the prognostic potential of the suggested risk-associated *GST* genotype combination. The frequency of genotype combination comprising three risk-associated genotypes was the highest in the group of patients with severe COVID-19. Consequently, in these individuals the OR to develop severe form of the disease was 4.7 times higher in comparison with carriers of reference genotype combination (Table 6).

**TABLE 6:** The association of risk-associated *GST* genotypes with the risk for severe COVID-19.

Number of risk-associated *GST* genotypes	Mild COVID-19 n (%)	Severe COVID-19 n (%)	OR (95%CI)^a^	*p-*value^c^
0	13 (17)	11 (8)	1^b^	
1	30 (40)	53 (40)	2.1 (0.83–5.24)	0.117
2	28 (37)	52 (39)	2.2 (0.87–5.54)	0.096
3	4 (5)	16 (12)	4.72 (1.22–18.39)	0.025

Mild COVID-19: Stage I; Severe COVID-19: Stages II + III + IV; 0: Reference genotype combination carrying lowest odds (*GSTP1-ValVal/GSTP1-ValVal*, *GSTM3-AA*); 1, 2, 3: The number of risk-associated alleles: either one, two or three risk-associated *GST*, genotypes (comprising GSTP1*Ile or GSTP1*Ala or GSTM3*C).

a OR, odds ratio adjusted for age, gender, hypertension, diabetes mellitus, smoking and obesity.

b Reference group; CI, confidence interval.

The results on the relation of the assessed *GST* genotypes with CT scan score, oxygen levels, C-reactive protein (CRP) concentration, and D-dimer and IL-6 levels in the patient's group are presented in Supplementary Table S1. No significant association was observed for any of the evaluated clinical manifestations of the disease and laboratory parameters. Interestingly, a certain trend was observed regarding CRP values which were the highest in carriers of variant *GSTM3-CC* genotype.

## Discussion

Based on the premise that oxidative stress plays an important role in SARS-CoV-2 infection, we speculated that variations in antioxidant activities of different members of the GST family of enzymes might modulate individual susceptibility towards the development of clinical manifestations in COVID-19. Among six *GST* polymorphisms analyzed in this study, *GSTP1* rs1695 and *GSTM3* rs1332018 were found to be associated with COVID-19. Indeed, the data obtained showed that individuals carrying variant *GSTP1*-*Val* allele exhibit lower odds of COVID-19 development, contrary to the carriers of variant *GSTM3*-*CC* genotype who have higher odds for COVID-19. Moreover, combined *GSTP1* (*rs1138272* and *rs1695*) and *GSTM3* genotype exhibited cumulative risk regarding both COVID-19 occurrence and COVID-19 severity. To our knowledge, this is one the first investigations that addressed the association of common GST polymorphisms and COVID-19.

Our results on the association of *GSTP1* polymorphisms with risk of COVID-19 seem biologically plausible since GST pi 1 (GSTP1) is highly expressed in lung tissue and might even be considered the predominant GST in the lungs (Terrier et al., 1990; Anttila et al., 1993; Cantlay et al., 1994; Rowe et al., 1997). Although GSTs seem to be ubiquitously expressed in human tissues, the expression of different GST genes may vary significantly between different tissues, giving each organ a unique and complex GST profile (Singh, 2015). Both genetic variants in *GSTP1* examined in our study, *Ile105Val* (rs1695) and *Ala114Val* (rs1138272), have functional relevance in terms of altered catalytic activity towards a variety of substrates and differences in GSTP1-mediated regulation of redox signaling pathways (Ali-Osman et al., 1997; Di Pietro et al., 2010). Apart from the extensive research on the role of polymorphic GSTP1 expression in various cancers, including lung cancers (Nørskov et al., 2017; Pljesa-Ercegovac et al., 2018; van de Wetering et al., 2021), several studies have also examined the association of these polymorphic variants with susceptibility or outcome in a range of communicable and non-communicable lung diseases (McMillan et al., 2016; van de Wetering et al., 2021). Specifically, in asthma, homozygosity of the *GSTP1 Val105* allele is associated with reduced risk of airway hyperresponsiveness and improved lung function (Fryer et al., 2000). Besides, genetic polymorphisms of *GSTP1* may be associated with chronic obstructive pulmonary disease (COPD) development suggesting GSTP1-*Val* allele to be more protective (Ishii et al., 1999). In a large population comprising 66,069 individuals, Norskov et al. showed that *GSTP1 Ile105Val* genotype was associated with improved lung function, with protection against lung cancer and tobacco-related cancer, as well as with reduced all-cause mortality. The findings were most pronounced in smokers for all end points, and they even suggested a gene dosage effect (Nørskov et al., 2017). Another lung disease in which GSTP1 inhibition is suggested as a novel therapeutic strategy is lung fibrosis, since GSTP1 is an important participant in protein S-glutathionylation (McMillan et al., 2016). Indeed, the immense group of cellular proteins, the so called “disulfide proteome” or the “glutathionome” might structurally and functionally be modified by glutathionylation (Lindahl et al., 2011; Pastore and Piemonte, 2012).

Despite the fact that *GSTP1* genetic variability might help our understanding of the susceptibility to COVID-19 disease, the data on the role of GSTP1 in SARS-CoV-2 are scarce. To our knowledge, this is the first case–control study conducted on COVID-19 patients that addressed the role of genetic polymorphisms in *GSTP1*. Namely, so far only one study based on the univariable analysis of the World Bank data showed that countries with more frequent *Val105* allele have higher prevalence and mortality of COVID-19 (Saadat, 2020b). However, our results on lower susceptibility towards COVID-19 development, obtained in COVID-19 patients, are in line with all the aforementioned studies that support the idea that *GSTP1 Val105* plays a protective role in lung function deterioration. One of possible explanations of differential susceptibility among carriers of different *GSTP1* alleles may be the variations in regulatory roles of GSTP1. It is noteworthy to mention that, apart from its well-established role in detoxification and antioxidant protection, GSTP1 also exhibits leukotriene synthase activity, thus influencing pulmonary and extrapulmonary manifestations of COVID-19 (Al-Kuraishy et al., 2021). Still, the data on the effect of different *GSTP1* genetic variants on LTC4 synthesis are lacking. On the other hand, there are plenty of data regarding the role of GSTP1 in MAPK signaling, with special emphasis on c-Jun-NH2-terminal kinase (JNK) (Board and Menon, 2013). Namely, JNKs are important kinases that are activated in innate immune responses to viral infection and stimulate the activity of several significant cytokines, including interleukins (IL-2, IL-4) (Hemmat et al., 2021). What is more, several SARS-CoV proteins, such as N protein, ORF6 encoded protein, and 3a and 7a proteins were shown to phosphorylate and induce JNK activity in different cell lines potentiating the suggested crucial role of JNK signaling pathway in SARS-CoV infection (Mizutani et al., 2005; Ye et al., 2008; Varshney and Lal, 2011; Fung and Liu, 2017; Hemmat et al., 2021). GSTP1 acts as a negative regulator of JNK kinase-dependent apoptotic signaling pathways via GSTP1:JNK1 protein:protein interaction. The complex dissociates in case of increased ROS content, which in turn leads to the association of GSTP1 into oligomers and JNK1 activation. Once activated, JNK1 induces a series of events, starting from the phosphorylation of its substrate, the transcription factor c-Jun, and resulting in apoptosis (Adler et al., 1999; Board and Menon, 2013). It is important to note that the catalytic activity of GSTP1 remains intact even when GSTP1 is engaged in protein:protein interaction, suggesting that the active site of GSTP1 does not participate in this process (Tew and Townsend, 2012). Based on our findings, it is tempting to speculate that carriers of variant *GSTP1* alleles *Val105* and *Val114*, which exhibit better JNK inhibition (Thévenin et al., 2011), are less prone to COVID-19 disease development.

Another signaling pathway clearly shown to be disrupted in COVID-19 and involved in hyperinflammatory response is Keap1/Nrf2 [Kelch-like ECH-associated protein 1/nuclear factor (erythroid-derived 2)-like2] pathway, recognized as a key regulator of cellular redox homeostasis. Namely, specific adaptive cytoprotective response, which includes changes in the Keap1/Nrf2 pathway, is activated when cellular levels of ROS and electrophiles are increased (Chartoumpekis et al., 2015; Basak et al., 2017). Interestingly, Nrf2 regulates *GSTP1* gene activation (Vasieva, 2011; Bartolini and Galli, 2016), while at the same time, GSTP1 is capable of forming protein complex with Nrf2, which helps stabilize Nrf2 and its further activity (Bartolini et al., 2015). Still, the functional relevance of GSTP1 polymorphic expression in terms of Nrf2 stabilization needs to be elucidated.

Regarding the role of GST *mu* class in lung inflammation, *GSTM1* polymorphism has been most extensively studied since its lack, or the *null* genotype, is highly prevalent in the population, and associated with increased risk of inflammatory lung diseases (Wu et al., 2012). Indeed, GSTs, and especially GSTM1, are shown to contribute to enzymatic antioxidant capacity, protecting lungs from cell-derived endogenous and inhaled oxidants (Rahman and MacNee, 2000; Rahman et al., 2006). However, in our case–control study no association between *GSTM1* genotype and COVID-19 development was observed. Instead, we found that *GSTM3*, known to be in linkage disequilibrium with *GSTM1*, meaning it might influence GSTM1 expression and possess overlapping substrate specificity with GSTM1 (Hayes and Strange, 2000; Hayes et al., 2005), significantly contributes to COVID-19 development. Decades ago, two *GSTM3* alleles, *GSTM3-*A and *GSTM3-*C, were identified based on the presence of an intronic recognition motif for the Yin Yang 1 (YY1) transcription factor which is known to have a fundamental role in normal biologic processes such as embryogenesis, differentiation, replication, and cellular proliferation (Inskip et al., 1995; Di Pietro et al., 2010). Namely, one mutation of the *GSTM3* gene generates a recognition factor for YY1. This might especially be important in the lungs, since evidence suggests that GSTs are important mediators of normal lung growth and their contribution to the development of lung diseases in adults may already start *in utero*, continuing through infancy, childhood, and adult life, potentially contributing to so-called early life susceptibility (van de Wetering et al., 2021).

Apart from GSTM1, with whom GSTM3 shares an amino acid sequence identity of about 70%, it has around 35% sequence identity with the alpha, pi, and theta GST classes, underlying its role in the detoxification of carcinogenic compounds (Patskovsky et al., 1999). Indeed, *GSTM3* has been dominantly associated with various cancers, and changes in its expression are shown to affect the progression of various tumors (Wang et al., 2020), although it has also been associated with COPD and lung disease in children with cystic fibrosis (Flamant et al., 2004; Çalışkan et al., 2015; van de Wetering et al., 2021). The possible explanation for the role of GSTM3 in maintaining redox homeostasis and its established responsiveness to oxidative stress, in both communicable and non-communicable diseases, might be in the fact that the expression of GSTM3 is regulated by the domain-containing protein 1 (NSD1), whose H_2_O_2_-induced suppression further leads to the reduction of GSTM3 levels through the *−63A/C* TATA box (Liu et al., 2005; Chu et al., 2014). In this line, it might be speculated that the presence of *GSTM3-CC* genotype in individuals with SARS-CoV-2 infection might significantly affect antioxidant capacity and consequential oxidative stress-associated mechanisms underlying COVID-19 development.

Another rather common deletion polymorphism in genes encoding for human cytosolic GSTs, present in approximately 20% of Caucasians, is *GSTT1* gene deletion which results in complete lack of GSTT1 enzyme activity (Wiencke et al., 1995). It is actually one of the *GST* polymorphisms so far associated with COVID-19. Namely, in his theoretical ecologic study, Saadat suggested that the countries with lower frequency of the *GSTT1-null* genotype exhibit higher COVID-19 mortality (Saadat, 2020a). In this line, just recently, Abbas et al. showed that COVID‐19 patients with the *GSTT1*-*null* genotype have higher mortality rates, while they found no association with susceptibility towards COVID-19 development (Abbas et al., 2021). The results of our case–control study are in agreement with this finding.

Last but not least, one single-nucleotide polymorphism in genes encoding common GSTs analyzed in our study is *GSTA1* polymorphism. It is represented by three apparently linked SNPs: −567TOG, −69COT, and −52GOA, resulting in differential expression with lower transcriptional activation of the variant GSTA1*B (-567G, -69T, -52A) than common GSTA1*A allele (−567T, −69C, −52G) (Coles and Kadlubar, 2005). Although the antioxidant role of GSTA1 is well established and certain structural homology between GSTA1 and GSTP1 explains why GSTA1 can also suppress JNK1 signaling in a similar manner as GSTP1 (Romero et al., 2006; Pljesa-Ercegovac et al., 2018), we did not find any association between *GSTA1* genotypes and COVID-19 development.

Taken together, our results on the association between certain *GSTP1* and *GSTM3* genetic variants and COVID-19 have shed some light on the involvement of genetic susceptibility in COVID-19 development. Further pointing to the multifaceted role of GSTP1 as the dominant glutathione transferase class in lungs are the results regarding the role of combined GSTP1 (rs1138272 and rs1695) and GSTM3 genotype in both COVID-19 occurrence and severity. However, further studies are needed to clarify the exact roles of specific glutathione transferases once the SARS-CoV-2 infection is initiated in the host cell. Taking into consideration the role of different GSTs in the regulation of redox signaling pathways, our results might even contribute to better identification of potential targets for novel drugs that might aid patient treatment in this pandemic.

## Data Availability

The datasets presented in this study can be found in online repositories. The names of the repository/repositories and accession number(s) can be found at: https://redcap.med.bg.ac.rs/, AntioxIdentification.
