# Supplementary material for: GSTP1 and GSTM3 Variant Alleles Affect Susceptibility and Severity of COVID-19
Source: Front Mol Biosci. 2021 Dec 20;8:747493. doi: 10.3389/fmolb.2021.747493 (PMC8721193; doi:10.3389/fmolb.2021.747493)
Supplement: Supplementary file 1 [file Table1.DOCX]

**Table S1. The association between *GST* genotypes and oxygen saturation, CT score, D-dimer, CRP and IL-6**

| ***GST* genotype** | **Oxygen saturation (%)**^c^ | **p-value** | **CT Score**^d^ | **p-value** | **D-dimer**  **(mg/L FEU)**^d^ | **p-value** | **CRP**  **(mg/L)**^d^ | **p-value** | **IL-6**  **(pg/ml)**^d^ | **p-value** |
| --- | --- | --- | --- | --- | --- | --- | --- | --- | --- | --- |
| *GSTM1* |  |  |  |  |  |  |  |  |  |  |
| *active^a^* | 95.4±2.9 |  | 10.5  (3-21) |  | 0.49  (0.18-4.17) |  | 33.7  (1.0-282.2) |  | 12.8  (1.5-213) |  |
| *null^b^* | 95.89±2.8 | 0.260 | 12.0  (1-20) | 0.255 | 0.47  (0.19-10.80) | 0.888 | 26.7  (0.5-245.8) | 0.484 | 26.0  (1.4-205.5) | 0.510 |
| *GSTT1* |  |  |  |  |  |  |  |  |  |  |
| *active^a^* | 95.7±2.9 |  | 12.0  (1-21) |  | 0.47  (0.18-10.80) |  | 29.7  (0.5-282.2) |  | 24.95  (1.4-213) |  |
| *null^b^* | 95.73±2.8 | 0.920 | 11.0  (4-18) | 0.422 | 0.50  (0.19-8.26) | 0.432 | 33.5  (1.1-164.4) | 0.742 | 14.30  (1.5-168.2) | 0.393 |
| *GSTA1 (rs3957357)* |  |  |  |  |  |  |  |  |  |  |
| *CC (active)* | 95.8±2.4 |  | 12.0  (3-16) |  | 0.49  (0.18-10.80) |  | 44.2  (0.6-282.2) |  | 28.30  (1.5-168.2) |  |
| *CT* | 95.6±3.1 |  | 11.0  (3-21) |  | 0.51  (0.19-2.12) |  | 32.4  (0.5-280.5) |  | 14.40  (1.5-213) |  |
| *TT* | 95.6±3.1 | 0.879 | 12.0  (1-18) | 0.947 | 0.45  (0.19-2.16) | 0.590 | 22.5  (0.9-224.5) | 0.267 | 27.90  (1.40-205.5) | 0.686 |
| *GSTP1 (rs1695)* |  |  |  |  |  |  |  |  |  |  |
| *IleIle (wild-type)* | 95.6±2.8 |  | 12.0  (4-20) |  | 0.50  (0.19-8.26) |  | 27.85  (0.6-282.2) |  | 19.55  (1.4-168.2) |  |
| *IleVal* | 95.7±±2.8 |  | 13.0  (1-21) |  | 0.46  (0.18—10.80) |  | 36.2  (0.5-280.5) |  | 17.0  (1.5-213) |  |
| *ValVal* | 94.7±3.7 | 0.418 | 13.0  (9-17) | 0.518 | 0.46  (0.22-1.16) | 0.658 | 34.4  (2.5-187.5) | 0.615 | 57.9  (13-115.1) | 0.244 |
| *GSTP1 (rs1138272)* |  |  |  |  |  |  |  |  |  |  |
| *AlaAla (wild-type)* | 95.9±2.6 |  | 12.0  (1-21) |  | 0.46  (0.18-10.80) |  | 31.5  (0.5-282.2) |  | 20.8  (1.4-196.9) |  |
| *AlaVal* | 94.9±3.5 |  | 12.0  (3-18) |  | 0.51  (0.19-8.26) |  | 35.2  (1.0-158.8) |  | 33.9  (1.5-213) | 0.299 |
| *ValVal* | 98.0 | 0.121 | - | 0.561 | 0.48  (0.18-10.80) | 0.300 | 5.0 (-) | 0.567 | - |  |
| *GSTM3 (rs1332018)* |  |  |  |  |  |  |  |  |  |  |
| *AA* | 95.3±3.1 |  | 13.0  (3-20) |  | 0.43  (0.19-8.26) |  | 20.5  (0.5-245.8) |  | 12.9  (1.5-205.5) |  |
| *AC* | 96.3±2.1 |  | 12.0  (4-21) |  | 0.49  (0.18-1.28) |  | 33.8  (1.0-280.5) |  | 22.0  (4.0-196.9) |  |
| *CC* | 95.7±3.0 | 0.188 | 12.0  (1-20) | 0.438 | 0.51  (0.19-10.80) | 0.349 | 46.9  (1.0-282.2) | 0.283 | 30.6  (1.4-213) | 0.597 |

*^a^Active*, if at least one active allele present; *^b^Null* if no active alleles present; ^c^Mean ±SD; ^d^Median (Min-Max)
